# Supplementary material for: Elevated olivine weathering rates and sulfate formation at cryogenic temperatures on Mars
Source: Nat Commun. 2017 Oct 17;8:998. doi: 10.1038/s41467-017-01227-7 (PMC5647339; doi:10.1038/s41467-017-01227-7)
Supplement: Supplementary file 1 — Supplementary Information [file 41467_2017_1227_MOESM1_ESM.pdf]

Supplementary Table 1. San Carlos Olivine -40 C

Exp #	Exp duration (d)	Exp duration (s)	Sample Mass (mg)	Surf Area (cm2/g)	Soln conc (Mg ppm)	Soln conc (Fe ppm)
1	9.13	788400	50.5	0.481	16.21	3.318
2	7.13	615600	50.6	0.481	14.87	3.078
3	5.13	442800	49.8	0.481	11.09	2.231
4	3.21	277200	50.6	0.481	9.82	1.927
5	2.13	183600	50.4	0.481	7.90	1.47
6	1.13	97200	50.4	0.481	5.48	0.902
7	0.88	75600	49.5	0.481	5.02	0.85
8	0.13	10800	50.4	0.481	4.17	0.593
9	0.04	3600	50.4	0.481	4.20	0.623
10	Blank	0	0	0	1.55	0.257

Supplementary Table 2. San Carlos Olivine -60 C

Exp #	Exp duration (d)	Exp duration (s)	Sample Mass (mg)	Surf Area (cm2/g)	Soln conc (Mg ppm)	Soln conc (Fe ppm)
1	14.00	1209960	50.00	0.481	6.95	1.343
2	9.01	778200	51.20	0.481	6.63	1.238
3	7.01	605700	50.10	0.481	7.25	1.737
4	4.85	418620	50.50	0.481	5.85	1.104
5	2.02	174120	50.40	0.481	6.07	1.139
6	1.05	90720	50.20	0.481	5.85	1.067
7	0.68	58740	49.50	0.481	6.15	1.159

Supplementary Table 3.
San Carlos Olivine, No acid -40° C

Exp day	Exp duration (d)	Sample Mass (mg)	Surf Area (cm2)	Soln conc (Mg ppm)	Soln conc (Fe ppm)
1/12/2012	14.00	50.40	0.481	1.17	NA
1/15/2012	11.01	51.00	0.481	1.10	NA
1/17/2012	9.01	49.40	0.481	1.07	NA
1/19/2012	7.01	49.80	0.481	1.12	NA
1/21/2012	4.80	49.40	0.481	0.96	NA
1/23/2012	3.01	50.20	0.481	0.92	NA
1/24/2012	2.02	50.90	0.481	1.04	NA
1/25/2012	1.05	49.50	0.481	0.88	NA
1/25/2012	0.68	49.20	0.481	0.89	NA
1/26/2012	0.02	49.60	0.481	0.73	NA

Supplementary Table 4.
No Olivine, acid + spheres, -40° C

Exp day	Exp duration (d)	Sample Mass (mg)	Surf Area (cm2)	Soln conc (Mg ppm)	Soln conc (Fe ppm)
7/23/2015	0	0	0	1.64	0.21
7/9/2015	1	0	0	1.40	0.26
7/9/2015	1	0	0	1.07	0.21
7/28/2015	7	0	0	2.46	0.19
7/28/2015	7	0	0	2.63	0.27

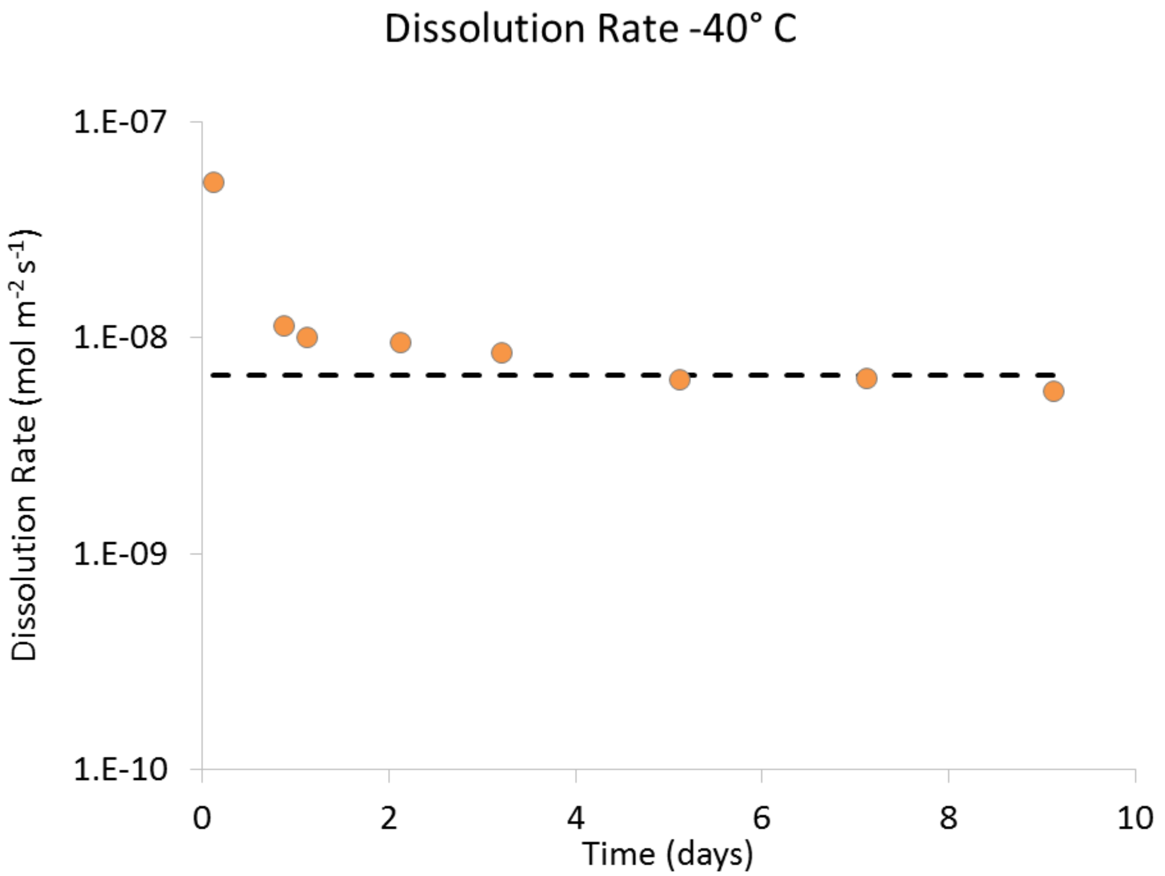

Supplementary Figure 1. Calculated dissolution rate of olivine over time. The black dotted line indicates the rate calculated from experiments with durations (>3 days). Uncertainties were calculated using standard deviation of the average.

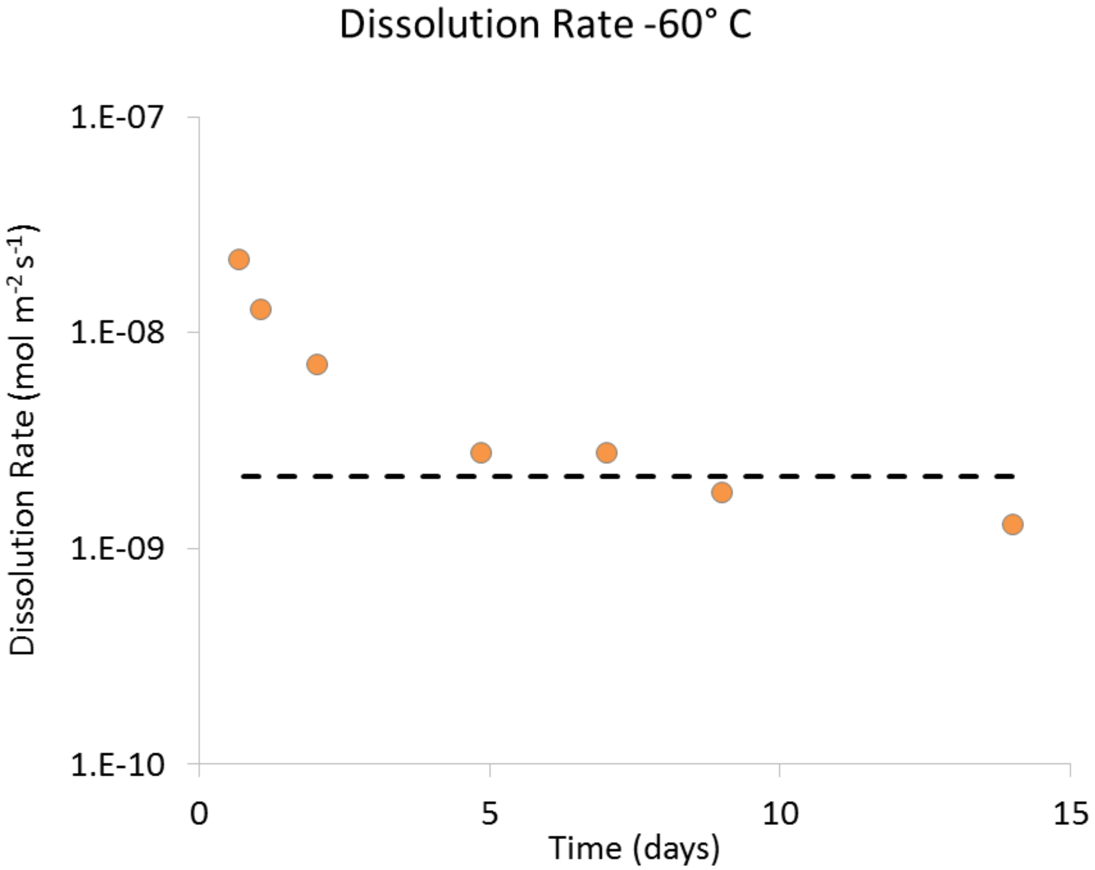

Supplementary Figure 2. Calculated dissolution rate of olivine over time. The black dotted line indicates the rate calculated from experiments with durations (>3 days). Uncertainties were calculated using standard deviation of the average.

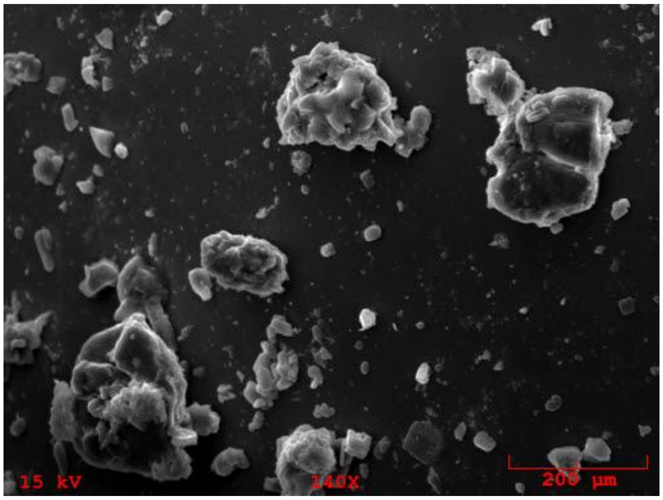

Supplementary Figure 3. SEM image of run products after 11 days at -60 C.

Spectrum	C	O	Na	Mg	Si	S	Ca	Fe
Image10_CGII-D-1	8.888	31.816	3.982	3.3	46.356	4.609	1.049	
Image10_CGII-D-2	7.092	31.051		19.273	10.849	27.701		4.033
Image10_CGII-D-3		34.847		21.572	2.918	36.784		3.88
Image10_CGII-D-4	2.367	27.707		19.959	5.111	39.216		4.9
Image10_CGII-D-5	0	30.154	5.566	17.779	4.874	39.317		
Image10_CGII-D-6		23.603		26.54	25.887	16.444		6.411
Image10_CGII-D-7			9.696	8.526	62.362	6.652	12.657	
Mean	4.587	29.863	6.415	16.707	22.622	24.389	6.853	4.806
Std. Dev.	4.113	3.847	2.95	8.014	23.458	15.145	8.208	1.16
Min	0	23.603	3.982	3.3	2.918	4.609	1.049	3.88
Max	8.888	34.847	9.696	26.54	62.362	39.317	12.657	6.411

Supplementary Table 5. EDS data are qualitative based on potential sample porosity and lack of surface smoothness. The EDS data do provide a demonstration that sulfur rich material was present and widespread in the solid run products. The presence of significant Na and Ca are derived from the Si-sphere which the grains are resting on and Si can be either from olivine, weathered silicate residue, or the Si spheres. Carbon is derived from carbon coating.
